# New Method for Analysis of the Temporomandibular Joint Using Cone Beam Computed Tomography

**DOI:** 10.3390/s21093070

**Published:** 2021-04-28

**Authors:** Sebastian Iwaszenko, Jakub Munk, Stefan Baron, Adam Smoliński

**Affiliations:** 1 GIG Research Institute, 40-166 Katowice, Poland; smolin@gig.katowice.pl; 2 Department of Temporomandibular Disorders, Medical University of Silesia, 41-800 Zabrze, Poland; jmunk@sum.edu.pl (J.M.); sbaron@sum.edu.pl (S.B.)

**Keywords:** cone-beam computerized tomography, temporomandibular joint, temporomandibular disorders, soft tissue thickness, image processing, image analysis, segmentation

## Abstract

Modern dentistry commonly uses a variety of imaging methods to support diagnosis and treatment. Among them, cone beam computed tomography (CBCT) is particularly useful in presenting head structures, such as the temporomandibular joint (TMJ). The determination of the morphology of the joint is an important part of the diagnosis as well as the monitoring of the treatment results. It can be accomplished by measurement of the TMJ gap width at three selected places, taken at a specific cross-section. This study presents a new approach to these measurements. First, the CBCT images are denoised using curvilinear methods, and the volume of interest is determined. Then, the orientation of the vertical cross-section plane is computed based on segmented axial sections of the TMJ head. Finally, the cross-section plane is used to determine the standardized locations, at which the width of the gap between condyle and fossa is measured. The elaborated method was tested on selected TMJ CBCT scans with satisfactory results. The proposed solution lays the basis for the development of an autonomous method of TMJ index identification.

## 1. Introduction

Imaging techniques in modern dentistry are increasingly based not only on classic radiology but also on magnetic resonance imaging (MR), conventional computed tomography (CT), cone-beam computed tomography (CBCT), and ultrasonography (USG). In the diagnosis of disorders involving the temporomandibular joint, cone-beam computer tomography, also known as volumetric tomography, plays a significant role because it allows a three-dimensional image to be obtained of nearly all of the head and neck calcified structures [1,2]. The conical shape of the X-ray beam enables the performance of the whole scan with only one rotation without sacrificing the resolution. The examination time can be reduced to several seconds [3], resulting in measurable benefits for both patients and dentists [1]. Among dental professionals, there are both opponents and supporters of this method; however, the vast majority finds it useful, which has been confirmed by numerous scientific studies analyzing the advantages of cone-beam tomography, as well as by the recommendations of scientific societies. The CBCT scans can be used for in vivo inspection of endodontic anatomy, which is important for treatment planning. The inspection of the scans reveals the number of roots and root canal system shape, previous treatments, and cavities, or confluences [4,5]. Volumetric tomography is considered to be a particularly useful method in the field of dentistry which deals with the dysfunction of the masticatory system; this results from the fact that it is more accurate and reliable than conventional tomography or pan-tomographic X-rays. It enables visualization of the bone components at high resolution in all dimensions. Application of CBCT can significantly reduce the radiation dose taken by the patient. It is markedly lower than when using traditional computed tomography or pan-tomographic X-ray images [6]. It also significantly reduces the examination time, which positively affects patients’ comfort. Although the dose in the case of some conventional methods (e.g., panoramic radiograms) can be lower than that absorbed during CBCT, the possibility of visualizing patient anatomy in 3D is lacking [3]. In addition to the above-mentioned advantages, this method has another important feature—it is cost-effective—both the price of the device itself and the cost of one scan are lower than in the case of competing methods (e.g., CT or MR). Due to its positive features, CBCT provides a wide range of applications in many other areas of dentistry, for example, dental surgery or orthodontics [7].

Despite unquestionable advantages of CBCT and its ability to depict the tiny details of the human body, the most important and most challenging part—the image interpretation—lies in the hands of experts. The interpretation task, as well as the visualization of particular structures in the volumetric data can be tedious and error prone. Therefore, suitable methods are being developed to address this issue. Among them, image processing constitutes one of the most promising ones. Image processing and further image analysis have found many applications in a variety of disciplines. Their numerous applications in industry [8,9], quality control, raw materials science [10,11], or in investigations of human behavior are widely known [12]. Needless to say, they are also commonly used in different aspects of medical science. Image processing and analysis are frequently applied in the identification of objects and structures visible on medical images. The detection of corneal endothelium cell borders on optical microscopy images can be mentioned as one of these examples [13]. There are also numerous applications in dentistry [14]. CBCT images have been used for research on trabecular jawbone quality assessment, which is one of the primary considerations during implant planning [15]. Quality is understood here as the density and structure of the bones. The authors focused on the appropriate bone segmentation on the CT images. The proposed method used adaptive thresholding, yielding satisfactory results. However, the research was targeted on inspection of bone structure. Though derived segmentation results were encouraging, it would be difficult to use it for macroscopic bone shape segmentation—the segmented images presented mainly the internal structure of the bones. The applicability of CBCT for the determination of selected material densities was examined by Pauwels [16]. A group of selected materials (air, aluminum, hydroxyapatite, polymethyl methacrylate) was tested in the phantom simulation of the human head. The materials were placed in various geometrical configurations, in order to study their mutual influence. The gray level values observed on CBCT scans were compared to the ones obtained with multislice computed tomography. Though the images obtained from CBCT were able to depict all the details, there were significant differences in observed gray levels for the same material in different configurations. It was therefore recommended that gray levels on CBCT images should not be used for qualitative measurements of a material’s density. The accurate calibration of grey values as HU is also hardly possible. However, even though the usage of the images for bone characteristic determination is questionable, the bone tissue can still be differentiated from other tissues with significant precision. The CBCT has been recognized as a useful tool for implantology. It was demonstrated, that CBCT along with an intraoral scan and an extraoral scan can be used for elaborating a 3D patient model [17]. The model was further used for designing implant placement. The CBCT imagery was also used in the development of a fully virtual articulator, enabling flexibility and treatment time reduction [18].

Attempts were made to use CBCT for measurement of the temporomandibular joint (TMJ) parameters. The position of the TMJ head, which can be determined by analyzing the space between the TMJ head and the fossa, is recognized as being most important [19]. There has been no full agreement between researchers regarding the optimal position of the condyle [20,21,22], some also having reported dependency between the shape of the condyle and its placement [23]. It is known, however, that disturbances in its position can cause many ailments. As examples, intra-articular pain, joint pain, or noises have been often mentioned [23,24]. A good example of the treatment of ailments caused by an anterior displacement of the disc in the temporomandibular joint is presented in [25]. Restoring the position of the disk, removed the symptoms, and the therapeutic effect was permanent. Imaging, in this case using nuclear magnetic resonance (MRI), was an important element of the therapy. Changing the positioning of the TMJ head in the glenoid fossa is a frequently chosen form of therapy. Therefore, a means of visualization and measurements of the condyle position before, during, and after the therapy is highly desirable. CBCT is considered to be one of the best techniques for that purpose [19]. However, the lack of automation of measurements of the TMJ head parameters has been observed. This may be caused by difficulties in segmenting the components of the temporomandibular joint, the lack of precisely (formally) defined methods of determining the reference plane based on which the measurements are made, or, finally, difficulties in determining the location of the points at which the slit width is measured. Despite experts being capable of precise and repeatable measurements, they are always prone to errors and subjectivity. The mentioned disadvantages have inspired research aimed at the development of an automated, image based method.

The aim of this research was to develop a method for determining the TMJ parameters on the CBCT scans. The proposed method allows the measurements to be performed in an almost fully automated way. The only part of the measurements, which has to be done manually is the selection of the volume of interest (VOI). Once the VOI is determined, all other operations can be performed automatically. As TMJ parameters are used in dentistry and orthodontics, increasing accuracy and consistency of their determination affect the effectiveness and comfort of treatment. The parameters sought are the measurements of the TMJ gap width with radiological fossa tomography in three locations: upper, frontal, and dorsal and evaluation of the condylar position in subjects with signs and symptoms of functional disorders of the temporomandibular joint through images made with cone-beam computed tomography on the sagittal plane [26]. Radiological fossa are the spaces without soft tissue, cartilage, and articular disc with bone borders. Their determination is performed in several steps. First, the highest point of the temporal fossa is determined. Then, the head of the TMJ is segmented and the method for measuring reference plane orientation is presented. Finally, the algorithm for the measurements of the parameters is described. The advantage of the proposed method is its simplicity: all operations can be done on a typical computer set in a short time, not exceeding one or two minutes. The present study proves that it is possible to measure the crucial parameters of TMJs and lays the foundation for a fully automatic analysis.

## 2. Materials and Methods

Presented retrospective research used the data gathered during the medical treatment of patients at the dental clinic at the Medical University of Silesia. All the data were anonymized before usage. The data are available for retrospective research for the University scientists.

The input data for the TMJ parameters consisted of 36 tomographic images. The scans were taken while ensuring that the patient’s head was in the Natural Head Position (NHP) [27], which is the standard orientation for CBCT scan acquisitions. The research was based on CBCT scans collected during normal medical treatment, taking place in the dental clinic of the University. The only criterion for image selection was the good quality of the scan (assessed by medical staff) and proper orientation of the head. The scans were taken between the years 2017 and 2020. The analyzed data were gathered using various devices (see Table 1). The images were initially processed by the native software provided by the manufacturers of CBCT devices to estimate the pseudo-HU values for the voxels. After that, all scans were saved as volumetric files in a Digital Imaging and Communications in Medicine (DICOM) compliant data format. The 3D images prepared in this way constituted the input data for determining the parameters of the TMJ.

**Figure 1:**
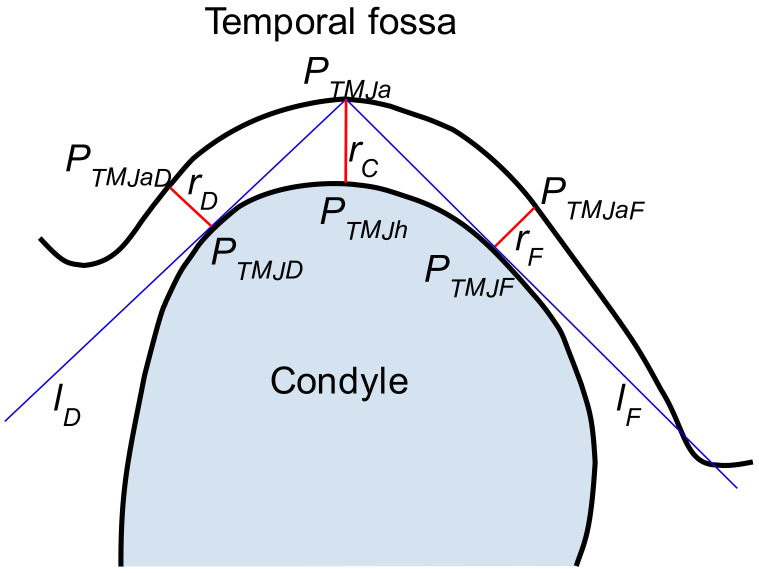
The schema of TMJ parameters determination.

The TMJ can be characterized by measurements of the parameters of the radiological gap between the ossified (calcified) structures including bones and in some cases during the inflammation process of cartilage. As is known from the literature, it is sufficient to measure the gap width in three carefully selected points [28]. The locations of these points, designated as *P_TMJD_*, *P_TMJh,_* and *P_TMJF_* are shown in Figure 1. The figure presents the cross-section through TMJ with the vertical plane, perpendicular to the TMJ head longer axis. The *P_TMJh_* is the highest point of the TMJ head. The *r_C_* is measured between it and that placed vertically above the point of the acetabulum, *P_TMJa_*. The *r_C_* is perpendicular to the Frankfurt Plane (FHP) [28,29].

The other two distances are measured on both sides of the TMJ head, one in the dorsal (*r_D_* = $\left\|\bar{P_{TMJD}P_{TMJaD}}\right\|$) and one in the frontal (*r_F_* = $\left\|\bar{P_{TMJF}P_{TMJaF}}\right\|$) position. The distances are measured at points *P_TMJD_* and *P_TMJF_* which are defined as the points where the lines *l_D_*, *l_F_* passing through *P_TMJa_* are tangent to the TMJ head outer shape. The perpendicular lines exposed at *P_TMJD_* and *P_TMJF_* intersect the acetabulum at *P_TMJaD_* and *P_TMJaF_*. However, it is significant in knowing how the section presented in Figure 1 is obtained.

**Figure 2:**
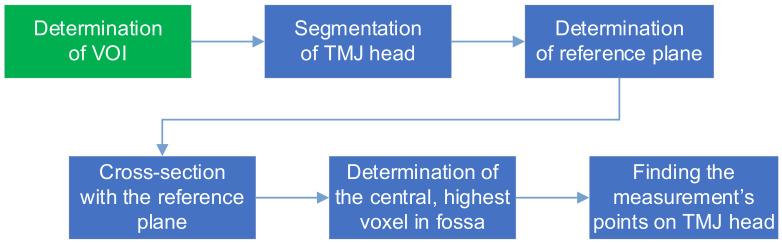
The determination of TMJ parameters workflow.

The following sections present the proposed method for the determination of the TMJ parameters upon the CBCT scan. The workflow of the process is presented in Figure 2.

The image analysis starts with preprocessing and VOI (Volume of Interest) identification. The VOI is a cuboid containing the TMJ. All further steps are limited to the VOI. The essence of determining the width of the TMJ gap is to ensure that the points at which this width is determined are unambiguously located. The most important thing is to eliminate the variability always present when the measurements are done by the physician. The proposed method deals with this problem by calculation of the TMJ cross-sectional plane, in which the measurements are then carried out, as well as determining the measurement points. The calculations are composed of set of well-defined steps, which are described in detail in the following sections.

The position of the section plane on which the measurements are made is characterized by the following conditions (called reference plane). The plane is vertical and is perpendicular to the plane containing a segment connecting the most distant points of the TMJ head. The plain containing the longest segment contained in the TMJ head also contains its horizontal projection, and the projection is normal to the sought reference plane. Determination of the reference plane should therefore start with identification of the projection of the longest segment contained in the TMJ head on the vertical plane. Therefore, the TMJ head is segmented on the CBCT using axial slices of the VOI. Once the TMJ head is segmented, the projection of the cross-section is determined, upon which the placement of the reference plane is calculated. In the next step, the vertical reference slice is determined and the *r_C_*, *r_D_*, and *r_F_* distances are obtained. The detailed descriptions of the proposed algorithm steps are presented in the following sections.

### 2.1. Preprocessing

**Figure 3:**
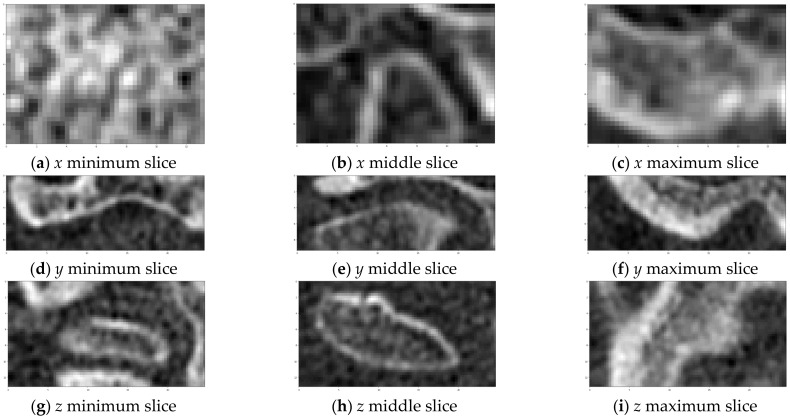
Example of VOI selection. The slices were taken on both sides and in the middle of VOI in each direction.

The CT scans produced by cone-beam tomography are known for their relatively high noise levels [1]. Therefore, noise reduction is one of the two procedures performed during the image preprocessing phase. There are many methods available for image denoising. A comprehensive review can be found in [30,31,32]. Inter alia, the curvature flow filter was chosen for application [33,34,35,36]. The filter is an example of an anisotropic diffusion filter [37] which allows smoothing of the image while preserving the edges [38]. The parameter values of the filter (time steps and the number of iterations) were determined by trial and error. The second of the preprocessing steps involved assessing the VOI. The step was performed manually using the slices view of the axial cross-section. First, the range of the *z* coordinate was determined. The highest *z* value should allow containment of the slice depicting the bone right above the TMJ acetabulum. The lowest point is the point where the analysis of the TMJ head should stop. The ranges of the *x* and *y* coordinates should be selected so that the TMJ head can be completely included inside the VOI leaving a little space on each size. The example of VOI selection is presented in Figure 3.

### 2.2. TMJ Head Segmentation

**Figure 4:**
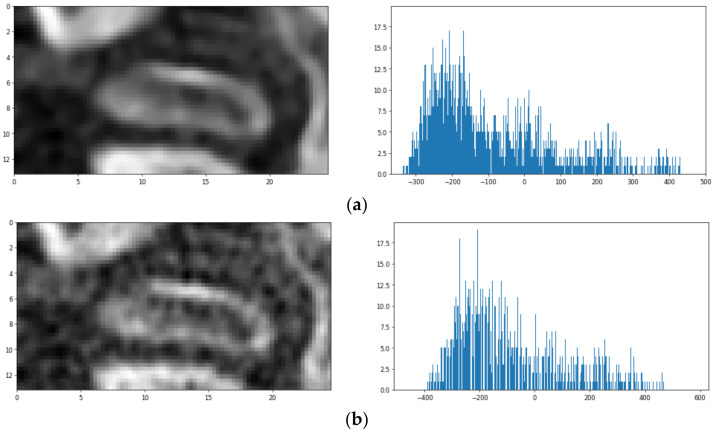
The example of axial slices (**a**) raw and (**b**) denoised and their histograms.

The identification of the TMJ head is based on a series of visualizations showing the patient’s skull in axial cross-section. The research was limited to the previously determined VOI region (becoming the Region of Interest—ROI on each slice). A series of views of consecutive cross-sections through the ROI containing the TMJ is presented in Figure 4. The initial assumption of the proposed algorithm is that the first (the uppermost) cross-section does not include any part of the TMJ acetabulum. In each slice, parts showing the bone have to be segmented. The segmentation is hampered by the fact that the CBCT image is usually noisy, the bones have a complex structure (they contain many voids) and the value of the voxels can significantly differ from the HU scale. Upon studying the set of images, the following observations were formulated:

- The pseudo-HU values in the vicinity of the TMJ are more representative of the voids (air) than the bones, hardly exceeding 200.
- The values differ significantly from pixel to pixel. However, the gradient at the edge of the fossa opening is significantly larger than within the bone or soft tissue.
- There are many voids visible which are separated by thin bone walls. They differ from the acetabulum in shape and size.

The observations allowed formulation of the TMJ head segmentation method. 

**Figure 5:**
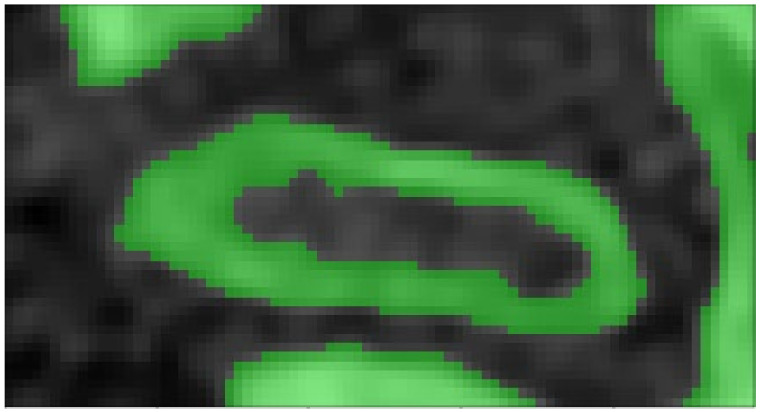
The first stage of segmentation (after GMM).

Let *S_i_* denote the *i*_th_ slice in the VOI. The slices are numbered from the superior towards the inferior and are perpendicular to the *z*-axis. Considering the histogram *h_i_* of the voxel values within the *S_i_*, it can be noticed that there is no apparent voxel value at which the image can be thresholded to achieve the requested segmentation. It can also be seen that the image after smoothing has a more compact histogram—almost all values within the range are present. In the histogram, one can observe a single big peak, resembling the Gaussian curve. The peak starts from the lowest values and spreads until approximately −100. The part of the histogram to the right of this value is rather flat, with two or three faintly visible peaks. The pattern can be observed in the vast majority of the analyzed slices. Only the slices showing the bone just above the acetabulum (*S*_0_, *S*_1_) have more uniform histograms, although the left-most peak is still clearly visible on them. The idea of segmentation is based on using the Gaussian Mixture Model [39,40]. The model approximates the given distribution with a set of Gauss distributions. The parameters of Gaussians are determined using the EM algorithm [41,42]. The best results using GMM (Gaussian Mixture Model) were obtained where each slice was used separately for model training. An example of the achieved segmentation can be seen in Figure 5.

It is apparent that the visible parts of bone tissue were correctly recognized. Despite that, some problems still need to be resolved. First, the TMJ head is not the only object made of bone tissue segmented in the image, which influences further steps of the analysis. On the other hand, the TMJ head is not segmented perfectly, i.e., there is a hole visible inside the TMJ head (as if the interior of the bone had a lower optical density); in some of the slices, the outer shape is not closed. Both of these drawbacks can be eliminated by further processing.

The elimination of head structures other than TMJ can be performed considering two insights. The other bone structures are placed at the borders of VOI and the condyle is the largest object placed in the central part of each slice image. The filling of the gaps in the middle of the TMJ head or closing its contours results from the assumption that its shape is not significantly different from its convex hull. Such an approximation does not affect the further analysis process. Therefore, this part of post-processing includes three steps. First, all segmented parts adjacent to the edges of the image are removed using morphological operations [43]. Second, all those remaining are replaced with their filled convex hulls. Third, all connected components are identified and labeled. Then, only the largest one is kept, as the TMJ head. After postprocessing, a properly segmented TMJ head is obtained, and further steps toward the TMJ parameters determination can be taken.

### 2.3. Determination of Reference Cross-Section Plane

In order to obtain repeatability of measurements with maximum accuracy, the authors propose a reference cross-section, which is the cross-section of the mandibular head along its long axis. This cross-section is considered along the axis close to the sagittal axis. This is because the mandible head is turned towards the center of the head. As a result of segmentation, a collection of slices (axial cross-sections) of the condyle is obtained. The next step involves finding the direction of the vertical section used for the determination of the TMJ parameters. The idea behind it is to find the longest section connecting the opposite points in the volume of the TMJ head. The projection of the section on an axial plane determines the orientation of the slicing vertical plane. The algorithm is constructed as follows: for each axial slice *S_i_*, containing the images of the segmented TMJ head, a minimal rectangle comprising the segmented area is constructed. Then, the shorter edges of the rectangle are considered. For each of the edges, the common points between them and the segmented area are identified. If there is more than one point common to the edge, the mean is used as the selected common point. For each slice *S_i_*, there are two points determined in that way. The comparison of the points along with the slices allows the ones most spread out to be selected. The section connecting them is the sought after longest section. Once the section is determined, its perpendicular bisector defines the section plane orientation. As the section plane is vertical, the knowledge of the bisector is sufficient for appropriate TMJ slicing. The formal description of the algorithm is presented in Algorithm 1.

The points *P*_0_, *P*_1_ are defined in a 2D space. They are the ends of the longest section projection on the axial plane. The sought cross-section plane $\pi_{V}$ is vertical and perpendicular to the $\bar{P_{0}P_{1}}$ section, crossing it in the middle. The slice *S^V^* represents the base slice for estimating TMJ parameters.
**Algorithm 1.** The longest section search algorithmMAX_I = height(VOI) #search is performed within VOI only i = i_0_ # i_0_ is the index of the first slice containing the TMJ head p0_max_lst = [] # list, beginning point of the section p1_max_lst = [] # list, ending point of the section while i < MAX_I:      TMJ_head_contour = findTMJContour(*Si*)      rect = find_min_area_rectangle(TMJ_head_contour)      # get list of rect’s shorter sides coordinates      sides = get_shorter_sides(rect)      # find mid points of all sections where rect’s side passes      # through the TMJ head slice. p0_lst, p1_lst are list.      p0_lst = get_mids_of_sections(img, sides[0])      p1_lst = get_mids_of_sections(img, sides[1])      # find the longest section connecting both sides of the TMJ head      p0, p1 = find_longest_segment(p0_lst, p1_lst)      p0_max_lst.append(p0)      p1_max_lst.append(p1)      i = i + 1 # find the longest of the connecting sections # p0, p1 contain the coordinates of beginning and ending # of the longest section p0, p1 = find_longest_segment(p0_max_lst, p1_max_lst)

### 2.4. Determination of TMJ Characteristic Parameters

The determination of the TMJ parameters *r_C_*, *r_D_*, *r_F_* requires the data contained in the slice, defined in the previous section. The image analysis starts with the determination of the highest temporal fossa point coordinates. Since the cross-cutting vertical plane is placed at an arbitrary angle, usually it is neither parallel to the coordinate system planes nor to voxels directions. Therefore, obtaining *S^V^* is almost always connected with resampling. Segmentation of the bone tissue should be repeated for the slice. However, this time, no hole filling is necessary.

The determination of the *P_TMJa_* point starts with the determination of the top of the TMJ head. It is easily done as the TMJ head is already segmented. The highest (first) slice $S_{i_{0}}$ containing the TMJ head is sought. On the found slice, the projection of the vertical, reference cutting plane $\pi_{V}$ is determined. Then, let us consider the part of the projection contained in the segmented TMJ head area. Its center is taken as *P_TMJh_*, the coordinates of the top of the TMJ head, on the cross-section slice defined by the cutting plane $\pi_{V}$. We find *P_TMJa_* as the first point (and in practice voxel) assigned to bone tissue lying on a vertical line contained in $\pi_{V}$ and passing through *P_TMJh_*. The knowledge of *P_TMJa_* and *P_TMJh_* coordinates allows the *r_C_* parameter to be calculated, as it is the length of the $\bar{P_{TMJa}P_{TMJh}}$ section:

(1) $$r_{C}=\|\bar{P_{TMJa}P_{TMJh}}\|$$

**Figure 6:**
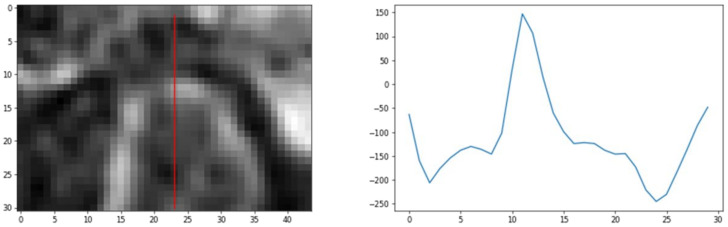
The CBCT image slice with a profile line in red (**left** image), and the voxels values across the line (**right** image).

The determination of *r_F_* and *r_D_* requires finding of the lines passing through the *P_TMJa_* and tangent to the TMJ head (see Figure 1). The lines are determined using the idea of the value profile. The given line is interpreted as a one-dimensional coordinate axis with an arbitrarily selected origin point. The profile of the value of the voxel is understood to be the function whose support is defined as the selected line passing through the volume of the CBCT image. The function maps the points on the line to the values of voxels crossed by the line. An example of the profile is presented in Figure 6.

**Figure 7:**
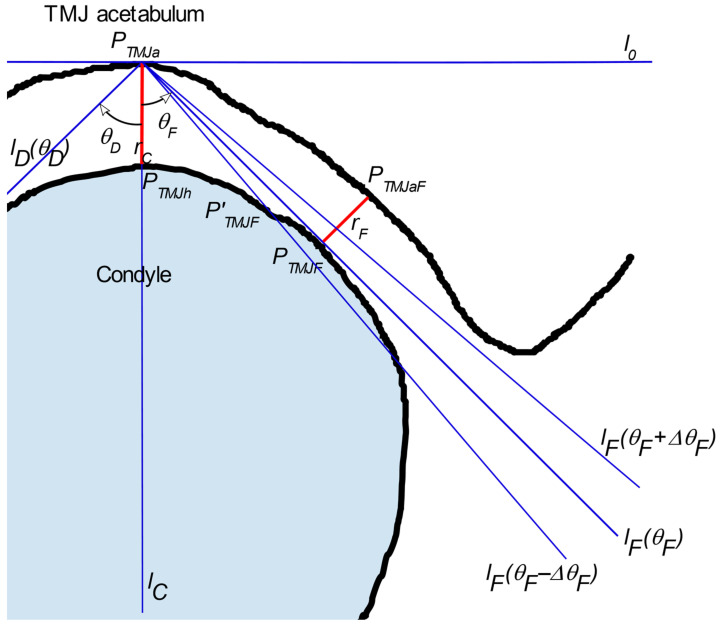
Determination of the line tangent to the TMJ head.

Let the *l_C_’* be the vertical line passing through the points *P_TMJa_* and *P_TMJh_* (the *r_C_* is included in this line). Let us define the lines *l_D_’* and *l_F_’* as the lines lying on the plane *π_V_* defined by the vertical slice and passing through the point *P_TMJa_*. Let the lines be different from line *l_C_* and different from each other. Let the *θ_D_* and *θ_F_* be the angles between *l_D_’* and *l_C_’* and *l_F_’* and *l_C_’*, respectively (see Figure 7). The half-plane *π*’*_V_* defined by line *l_o_* and the part of plane *π_V_* lying beneath it are considered. Let the lower parts of the *l_D_’*, *l_F_’,* and *l_C_’* lines starting at *P_TMJa_* be the half-lines *l_D_*, *l_F_* and *l_C_* respectively. The line *l_o_* is orthogonal to the line *l_C_’* and passes through *P_TMJa_*. The half-lines *l_D_*, *l_F_* lie on the opposite sides of the *l_C_*. Let Δ*θ* be the assumed step in which the *θ* angle will be changed. As the angles *θ_D_* and *θ_F_* are measured in opposite directions, let us use Δ*θ_D_* and Δ*θ_F_* to represent angle increment in the *θ_D_* and *θ_F_*, respectively. The directions of the Δ*θ_D_* and the Δ*θ_F_* are the same as the angles *θ_D_* and *θ_F_*. Considering the *l_F_* line, the tangent searching procedure is described as follows: let the angle *θ_F_* change according to the following rule:

(2) $$\theta_{F}^{0}=\Delta \theta_{F}\;;\;\theta_{F}^{i+1}=\theta_{F}^{i}+\Delta \theta_{F}$$

**Figure 8:**
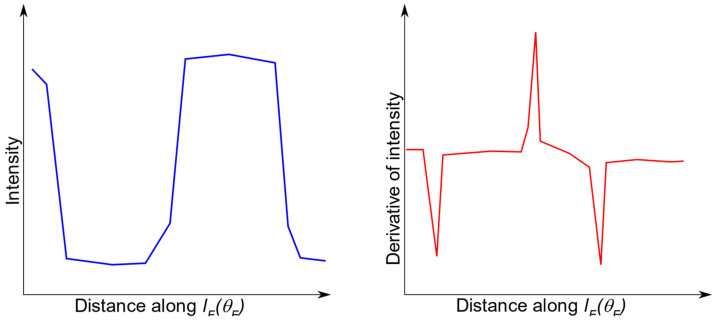
Expected line profile (**left** image) and its first derivative (**right** image). The distance coordinate starts at PTMJa, the line passes the border between bone and soft tissue three times: leaving the acetabulum (first derivative peak, negative), entering the TMJ head (second derivative peak, positive), and leaving the TMJ head (third derivative peak, negative).

The direction of line *l_F_* depends on the selected value of the *θ_F_*, *l_F_ = l_F_ (θ_F_)*. Similarly, the direction of the *l_D_* depends on the selected value of the *θ_D_*, *l_D_ = l_D_ (θ_D_)*. Let *P_TMJD_* and *P_TMJF_* denote the points where lines *l_D_* and *l_F_* are tangent to the TMJ head. Next, let *P’_TMJD_* and *P’_TMJF_* be the points where the lines *l_D_* and *l_F_* cross the TMJ head (Figure 7). Further analysis is focused on one of the lines, *l_F_*, for clarity. The reasoning for the other line, *l_D_*, can be carried out similarly. Let us construct 2 profiles for *θ_F_^i^* and *θ_F_^i+^*^1^ angles, *l_F_ (θ_F_^i^),* and *l_F_ (θ_F_^i+^*^1^*)*. Then, let each of the profiles be limited to the section contained in ROI. In another step, a derivative for each profile should be calculated. Depending on the angle *θ_F_*, the line *l_F_ (θ_F_)* crosses the boundary between bone and the soft tissues at least once. Therefore, the derivative should contain at least one peak in the places where the profile passes between the gap in the acetabulum and the bone. The first peak is placed in the *P_TMJa_*. If the *θ_F_^i^* is small enough, the second peak is located at the point where *l_F_* crosses the TMJ head (*P’_TMJF_*). The third (and further) peak can be observed if *l_F_* crosses the TMJ head boundary once again, going from bone to soft tissues. At least one of the peaks has a negative value, while the most interesting one, if present, has values greater than zero (Figure 8).

Let us consider the length of the section $\left\|\bar{P_{TMJa}P_{TMJF}^{\prime }}\right\|$. It is worth noting that *P’_TMJF_* is dependent on the angle *θ_F_*, *P’_TMJF_ = P’_TMJF_ (θ_F_)*. With the increase of the *θ_F_^i^*, the length of the section also increases, as the *l_F_* approaches the tangent to the TMJ head. Let *θ_F_^T^* denote the angle at which the line *l_F_* is tangent to the TMJ head. As the *θ_F_* becomes greater than *θ_F_^T^*, the line *l_F_* no longer crosses the TMJ head (consider the lines *l_F_ = f(θ_F_^i^)* and *l_F_ = f(θ_F_^i+1^)* as shown in Figure 7). The placement of the second peak in the line profile’s derivative appears when the line again passes the soft tissue/bone border. However, this time, the line no longer passes through the TMJ head and the border is located significantly further than the $\left\|\bar{P_{TMJa}P_{TMJF}^{\prime }}\right\|$ observed for the *l_F_ = f(θ_F_^T^).* It is also possible that there is no soft tissue to bone crossing within the ROI. Observing the distance between first and second peaks visible on the profile derivative as the function of *θ_F_*, the discontinuity (step) can be noticed as *θ_F_* exceeds *θ_F_^T^*. Once such a step is detected, its placement reveals the *θ_F_^i^* for which the line *l_F_* becomes tangent to the TMJ head. The placement of a derivative peak on the profile determined for such an angle shows the placement of the sought point *P_TMJF_*. Because the numeric algorithm assumes that *θ_F_* is incremented with step Δ*θ_F_*, further refinement of the *P_TMJF_* position is possible. For that, it is suggested that binary search within the angle values section *<θ_F_^i^, θ_F_^i+^*^1^*>* should be performed. Alternatively, Δ*θ_F_* can be chosen small enough to ensure the requested precision.

Once *P_TMJF_* is determined, the *r_F_* value can be easily calculated. At the point defined by the peak (*P_TMJF_*), a line placed on the plane *π_V_* and perpendicular to the tangent *l_F_(θ_F_^T^)* can be drawn. Let the point *P_TMJF_* be the origin on this line and the coordinate system be directed towards the acetabulum. The distance between point *P_TMJF_* and the crossing point is the sought *r_F_*. The analogous procedure should be performed for the other side of the *l_C_*, resulting in the determination of *r_D_*. The tuple (*r_F_*, *r_C_*, *r_D_*) forms the parameters describing uniquely the TMJ.

## 3. Results and Discussion

The assessment of the proposed method was twofold. First, the ability of appropriate identification of the objects sought at each step was checked. Second, the comparison with the results achieved by experts was carried out. The first stage involves the testing of the robustness of the algorithm itself. What kind of difficulties occur during the process is investigated. Therefore, the used input data (CBCT images) were collected using various devices (CBCT tomographs). The experiments were carried out as follows. First, the preprocessing stage was performed. All values in the DICOM files were transformed into HU units. The VOI for each TMJ was manually determined, as described in the previous section. The VOI was remembered as the *x*, *y*, and *z* coordinate ranges. After the VOI was identified, the noise level was limited by the application of the curvature flow filter. The best results were obtained when the time step for the filter was equal to 0.01 and 10 iterations were performed. After this, the preprocessed images with defined VOIs were ready for further processing.

### 3.1. TMJ Head Segmentation and π_V_ Slice

**Figure 9:**
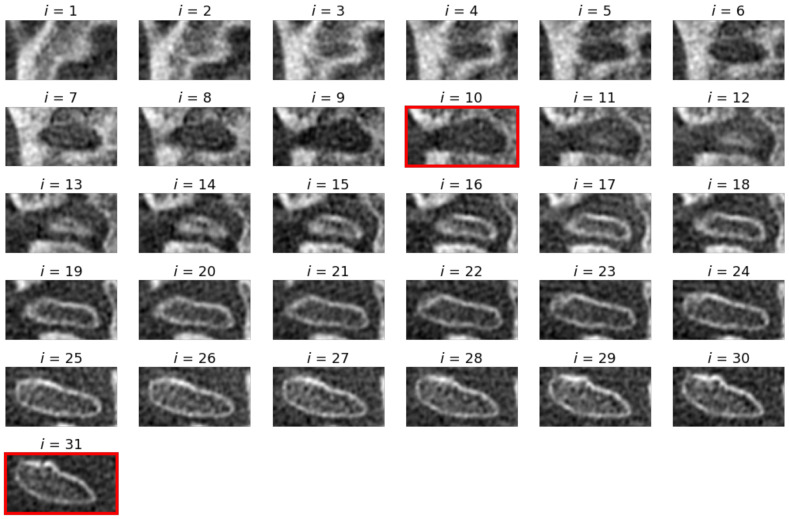
The consecutive slices of input scan restricted to VOI. The first and last slices with identified TMJ head bone tissue are marked with red frames.

**Figure 10:**
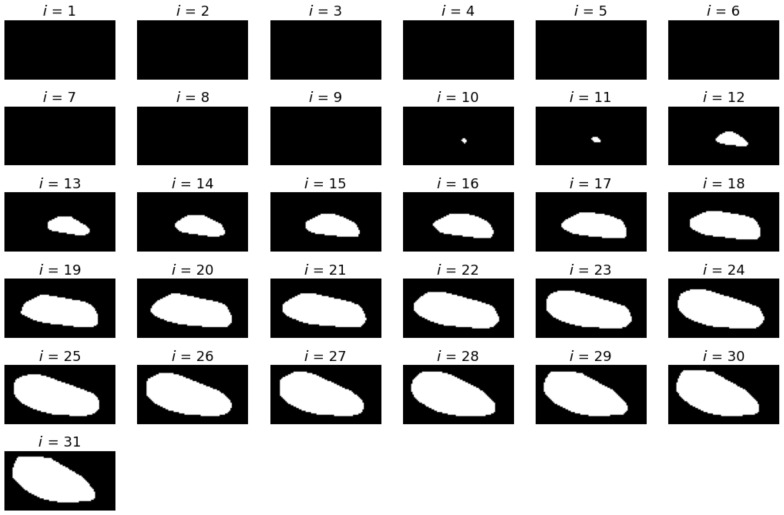
The slices containing the TMJ head segmented from the slices presented in Figure 9. The first pictures are black, as no part of the TMJ head was present on them.

An exemplary set of axial slices images is presented in Figure 9. The slices are limited to VOI and constitute the input for further processing. The results of the TMJ head segmentation are visible in Figure 10. The head of the joint has been properly segmented. The shape of the head is a little bit thicker than might be expected looking at the source images. It is caused by approximating the head by its convex hull. However, the approximation does not influence the determination of the $\pi_{V}$, as the length of the TMJ head remains unchanged. For each of the shapes, the minimum area rectangle is determined and the longest section connecting the points lying on the opposite sides of the rectangle is calculated. For that, the sets of common pixels between the shorter sides of the rectangle and the TMJ head shape are found. Then, all possible sections connecting the points are determined. The coordinates of the ends of the longest ones are remembered for further processing. The process is repeated for each segmented axial slice section of the TMJ head. The ends of the sections are used for determining the direction of the $\pi_{V}$ plane. The plane is vertical and crosses the mid-point of the projection of the section connecting the innermost and the outermost voxel identified during the axial slices analysis.

**Figure 11:**
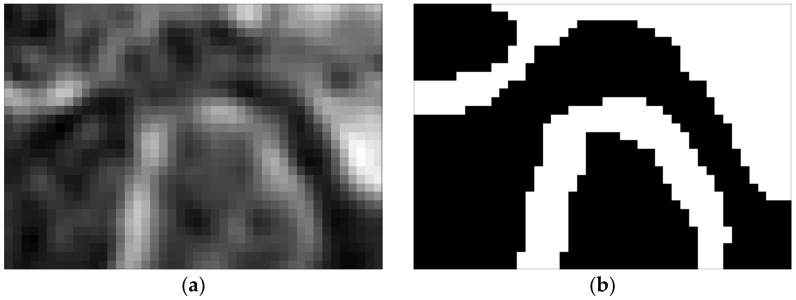
Reference slice used for the determination of TMJ parameters: (**a**) slice taken in *π_V_* plane and (**b**) slice segmentation.

This plane creates a certain angle with the *y* axis of the coordinate system. Knowing the position of the said section, one can easily find this angle. Determining the cross-section given by this plane requires finding of all the voxels it cuts through and interpolating their values. In practice, it turned out to be more convenient to rotate the entire scan by this angle so that the $\pi_{V}$ plane becomes parallel to one of the planes of the coordinate system. The reference cross-section obtained in this way is shown in Figure 11.

### 3.2. Determination of TMJ Parameters

**Figure 12:**
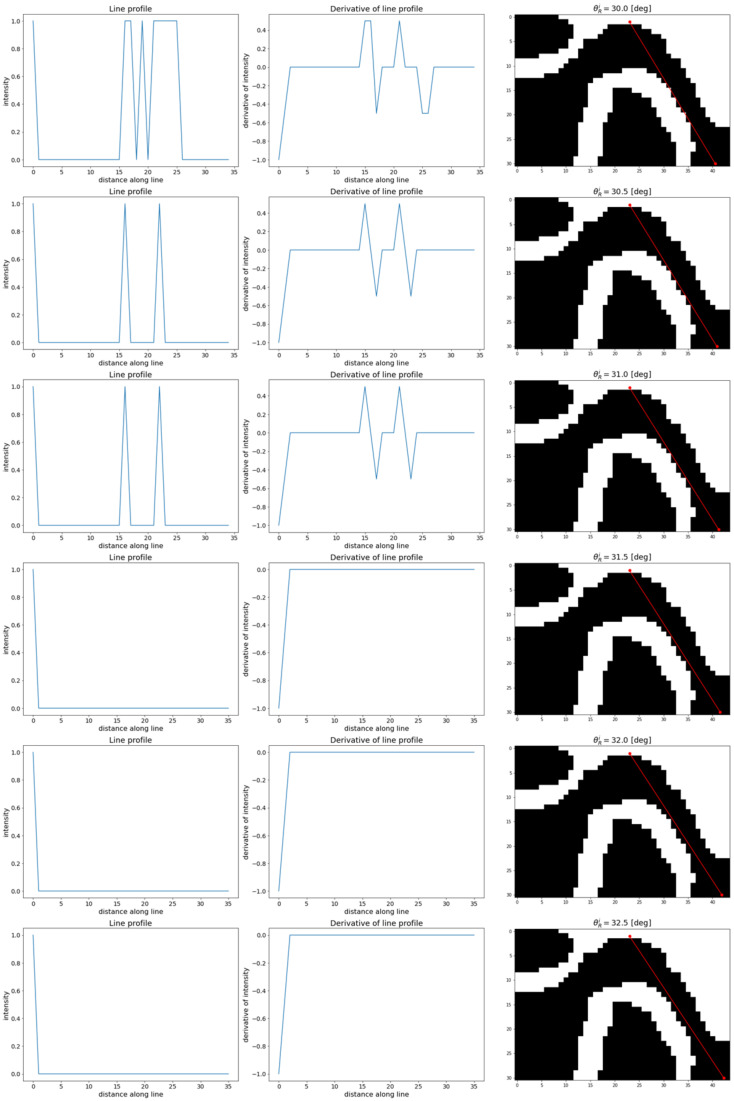
Iterative procedure results for the determination of the TMJ head tangent placement.

**Figure 13:**
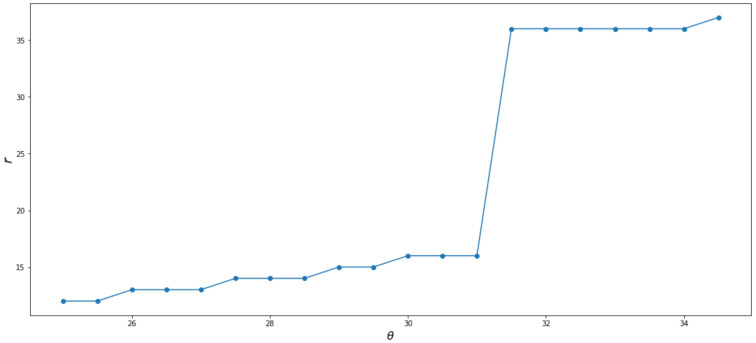
Distance to the first slope as function of *θ*.

All further analyses are performed on the reference slice. Determining the TMJ parameters requires finding the *P_TMJa_* point. First, the highest set of voxels on the TMJ head is determined. The vertical line is constructed in the middle of the identified voxels. The *P_TMJa_* is found as the first voxel on the segmented bone tissue lying on the constructed line. Once it is determined, the iterations start to find the tangent to the TMJ head. The procedure used was divided into two steps. In the first one, a rough scan of the profiles was performed. The *θ* angles were changed from 0° to 50° with 5° steps. Next, the angle interval was limited to the vicinity of the tangent placement, and the step was reduced to 0.5°. For each line orientation, a line profile and its first derivative were calculated. The results for the example data are presented in Figure 12. It can be seen that both the profile and the derivative of the profile follow the expected path. What is most important for the proposed procedure is the placement of the first slope representing the crossing from the soft tissue (the TMJ gap) to the bone (the TMJ head). Therefore, it is useful to consider the distance to the first slope as a function of *θ*. The function is presented in Figure 13. The distance grows slowly from the *r_C_* as the *θ_R_* increases. An abrupt step is clearly visible between *θ_R_* = 31° and *θ_R_* = 31.5°. The *θ_R_^T^* can be taken as the mean of the two values yielding 31.25°. The analogous procedure performed for the left side of the reference slice gives *θ_L_^T^* = −32.75°. The determination of distances *r_R_* and *r_L_* are straightforward as well as the lines perpendicular to the found *l_R_* and *l_L_* at points *P_TMJR_* and *P_TMJL_*. The profiles reveal the placement of the ends of *r_R_* and *r_L_*. The distances calculated for the presented example data gave the following values:

*r_L_* = 2.1 mm, *r_C_* = 3 mm, *r_R_* = 1.5 mm.

The reference points determined by the method are based on the method described by Ikeda and Kawamura [28]. The proposed enhancement focuses on two aspects: the method for unambiguous determination of the cross-section reference plane $\pi_{V}$ (proposed by J. Munk), and the algoritm allowing the application of the method, using the CBCT scan. The biggest advantage of the method is its unambiguity and independence of the human factor. The reference points are determined repeatedly and cohesively each time in the same place, conversely to the manual measurement. Though reliable manual measurements performed by experts have been often presented in [19,44], an automated method should be preferred. It is also advantageous to lower the time necessary for the parameters’ determination. The application of GMM as a tool for condyle segmentation proved its robustness. In contrast to the thresholding used in other research studies [45,46], the method is less influenced by deviation in voxel values. Though the proposed method performed well, certain conditions may contribute to its failure. The Utilized Gaussian Mixture Model and EM algorithm can fail if there is no sufficient difference between bone and soft tissue voxel values on the CBCT scan. In such circumstances, the segmentation method should use also information concerning the location and the vicinity of the segmented regions. There might be problems with the identification of the TMJ head shape in cases where its significant degeneration is observed. The same can cause problems with tangent identification. Having said that, it must be stated that these are rather exceptional cases.

The presented method assumes, that the patient’s head during the CBCT data acquisition is in NHP. Despite the stabilization of the patient’s head and performing the CBCT scan by experienced personnel, some deviation from the NHP can be detected. The proposed method will be influenced by it, regardless of the type of deviation. The usage of the large field of view, which is typical for TMJ scans, allows visualization of the points in the skull which can be used for the determination of the orientation of the Frankfurt plane [47], and can be used to correct the head position digitally, by applying appropriate rotations. It is strongly recommended that the head position is checked and corrected if necessary. The development of an automatic method for Frankfurt plane determination was also reported in [48].

The presented procedure proved to be successful in the determination of TMJ parameters. The method is deterministic and therefore tabulated and repeatable measurement results are obtained. The same set of CBCT scans lead to the same results, which is advantageous for the manually performed assessment. Once the VOI is determined, the method leaves no space for subjectivity. the procedure also opens up the possibility of determining the parameters independently of the doctor’s qualifications and predispositions. The good head position and determination of VOI seem to be the most important for the presented method. As the TMJ gap width measurement points are oriented to the highest point of the fossa, the incorrect position of the patient’s head will affect the robustness of the method. Once the VOI is properly established, the method will give consistent and reliable results. However, the VOI determination is not always obvious. The perfect VOI should contain both condyle and the fossa while including as few other adjacent bone structures as possible. Therefore, further research should aim at developing methods for an automatic assessment of the TMJ including VOI.

## 4. Conclusions

The article presents a method for determining the parameters of the temporomandibular joint based on CBCT scans. The joint is characterized by the values of the width of the gap between the condyle and the acetabulum, measured at three selected characteristic points. The proposed method enables the determination of these parameters in an automated manner, which has a positive effect on the consistency, repeatability, and reliability of measurements. The crucial steps of the method include segmentation of the condyle on the axial slices of the VOI and the determination of the reference plane. The reference plane defines the vertical cross-section used further for the TMJ gap widths. The well-defined algorithm for the reference plane determination and the identification of the measurements points forms a common foundation for measurements. Formalizing the process allowed for its automation. The presented algorithm is a new proposition for dental practice. However, the method can still be improved. For example, it can be enhanced with automatic VOI determination or head position correction. Despite this, the improvement in the time of measurements and the precision are readily detectable.

## Figures and Tables

**Table 1:** The CBCT devices and software used for imaging and analysis.

Device Type	Software Name	Vendor
Kavo OP3DPRO	InVivoDental	KaVo Dental Excellence
Morita X 800	i-Dixel	J. Morita Corporation
iCat	InVivoDental	KaVo Dental Excellence

## Data Availability

Data sharing not applicable.
